# Mutually exclusive mutations in *NOTCH1* and *PIK3CA* associated with clinical prognosis and chemotherapy responses of esophageal squamous cell carcinoma in China

**DOI:** 10.18632/oncotarget.6120

**Published:** 2015-10-29

**Authors:** Bin Song, Heyang Cui, Yaoping Li, Caixia Cheng, Bin Yang, Fang Wang, Pengzhou Kong, Hongyi Li, Ling Zhang, Zhiwu Jia, Yanghui Bi, Jiaqian Wang, Yong Zhou, Jing Liu, Juan Wang, Zhenxiang Zhao, Yanyan Zhang, Xiaoling Hu, Ruyi Shi, Jie Yang, Haiyan Liu, Ting Yan, Yike Li, Enwei Xu, Yu Qian, Yanfeng Xi, Shiping Guo, Yunqing Chen, Jinfen Wang, Guodong Li, Jianfang Liang, Junmei Jia, Xing Chen, Jiansheng Guo, Tong Wang, Yanbo Zhang, Qingshan Li, Chuangui Wang, Xiaolong Cheng, Qimin Zhan, Yongping Cui

**Affiliations:** ^1^ Translational Medicine Research Center, Shanxi Medical University, Taiyuan, Shanxi, China; ^2^ Key Laboratory of Cellular Physiology, Ministry of Education, Shanxi Medical University, Taiyuan, Shanxi, China; ^3^ Department of Oncology, The First Hospital, Shanxi Medical University, Taiyuan, Shanxi, China; ^4^ Department of Tumor Surgery, Shanxi Cancer Hospital, Taiyuan, Shanxi, China; ^5^ Department of Pathology, The First Hospital, Shanxi Medical University, Taiyuan, Shanxi, China; ^6^ BGI-Shenzhen, Shenzhen, Guangdong, China; ^7^ Department of General Surgery, the First Hospital, Shanxi Medical University, Taiyuan, Shanxi, China; ^8^ Department of Nuclear medicine, the First Hospital, Shanxi Medical University, Taiyuan, Shanxi, China; ^9^ Department of Pathology, Shanxi Cancer Hospital, Taiyuan, Shanxi, China; ^10^ Department of Endoscopy, Shanxi Provincial People's Hospital, Taiyuan, Shanxi, China; ^11^ Department of Statistics, Shanxi Medical University, Taiyuan, Shanxi, China; ^12^ School of Pharmaceutical Sciences, Shanxi Medical University, Taiyuan, Shanxi, China; ^13^ Key Laboratory of Medical Cell Biology, College of Translational Medicine, China Medical University, Shenyang, China; ^14^ State Key Laboratory of Molecular Oncology, Cancer Institute and Cancer Hospital, Chinese Academy of Medical Sciences and Peking Union Medical College, Beijing, China

**Keywords:** esophageal cancer, significantly mutated genes, mutational exclusivity, oncogene

## Abstract

**Background:**

Recurrent genetic abnormalities that correlate with clinical features could be used to determine patients' prognosis, select treatments and predict responses to therapy. Esophageal squamous cell carcinoma (ESCC) contains genomic alterations of undefined clinical significance. We aimed to identify mutually exclusive mutations that are frequently detected in ESCCs and characterized their associations with clinical variables.

**Methods:**

We analyzed next-generation-sequencing data from 104 ESCCs from Taihang Mountain region of China; 96 pairs were selected for deep target-capture-based validation and analysis of clinical and pathology data. We used model proposed by Szczurek to identify exclusive mutations and to associate these with pathology findings. Univariate and multivariate analyses with Cox proportional hazards model were used to examine the association between mutations and overall survival and response to chemotherapy. Findings were validated in an analysis of samples from 89 patients with ESCC from Taihang Mountain.

**Results:**

We identified statistically significant mutual exclusivity between mutations in *NOTCH1* and *PIK3CA* in ESCC samples. Mutations in *NOTCH1* were associated with well-differentiated, early-stage malignancy and less metastasis to regional lymph nodes. Nonetheless, patients with *NOTCH1* mutations had shorter survival times than patients without *NOTCH1* mutations, and failed to respond to chemotherapy. In contrast, patients with mutations in *PIK3CA* had better responses to chemotherapy and longer survival times than patients without *PIK3CA* mutations.

**Conclusions:**

In a genetic analysis of ESCCs from patients in China, we identified mutually exclusive mutations in *NOTCH1* and *PIK3CA*. These findings might increase our understanding of ESCC development and be used as prognostic factors.

## INTRODUCTION

Esophageal cancer causes 400,200 deaths each year and represents the sixth leading cause of cancer deaths worldwide [1]. It is categorized into esophageal adenocarcinoma and esophageal squamous cell carcinoma (ESCC) [1]. China has the highest incidence and mortality rate of ESCC in the world; approximately 70% of worldwide ESCC cases occur in China, where it is ranked the fourth most lethal cancer [2]. The risk of developing ESCC in China has been linked to factors such as gender, dietary habits, and family history. Alcohol abuse and tobacco consumption represent minor factors in high-risk populations in China [2]. Currently, the treatment of ESCC relies on surgery, chemotherapy, radiotherapy, or combinations of these [3]. Although many reports have indicated that patients who receive chemoradiation therapy had a significantly better 5-year survival rate than patients who did not, it is unclear why certain patients respond better than others to chemotherapy [3]. It is common for patients to be either under- or over-treated in China, which results in increased chemoresistance or treatment toxicity [4]. Therefore, unambiguous molecular markers are needed to identify which patients are likely to respond best to particular treatments, and thereby avoid under-treatment or over-treatment.

ESCC has a striking geographic distribution worldwide, with a higher prevalence in certain restricted areas of China [5]. Specifically, Shanxi, Henan, and Hebei provinces (the Taihang Mountains in North-Central China) are the so-called “esophageal cancer belt” with extremely high incidences of ESCC compared with the elsewhere in China and the rest of the world (Supplementary Figure 1); over 20% of all deaths in this area have been attributed to this cancer [5].

Epidemiological and etiological studies have shown that environmental and genetic factors play crucial roles in esophageal carcinogenesis [6]. Recently, genome-wide investigations have characterized genomic alterations in ESCC [7-9]. For these next-generation sequencing (NGS) studies, patients were enrolled from either Chaoshan District, Southern China [7] or the Cancer Institute and Hospital, Chinese Academy of Medical Sciences (CICAMS), where patients come from all over the country [8, 9]. Moreover, we previously performed an NGS study on 104 ESCC patients who were recruited from Shanxi and Henan provinces, combined with mutation set of 17 whole-genome sequencing (WGS) and 71 whole-exome sequencing (WES) from Chaoshan District and reported their mutational signatures and identified driver genes or pathways that contributed to ESCC [10]. In large collections of tumor samples, it has been observed that alterations that affect the same cancer pathways tend to not co-occur in the same patient. Mutual exclusivity may arise, because alteration of only one pathway component is sufficient to deregulate the entire process. Such mutually exclusive alterations have frequently been observed and have been associated with pathological and clinical features; thus, they can serve as molecular markers for potential categorization of tumors and prediction of the prognosis and treatment response of patients [11, 12]. Detecting such patterns is important for understanding cancer progression and prognosis and could suggest genes for targeted treatment or markers for the improvement of treatment in patients [13]. Some mutually exclusive alterations in the genomes of ESCC patients have been identified but not studied very much, and no reliable molecular markers for diagnosing or predicting patient outcomes have been identified for this disease [8, 9].

In this study, we describe the identification and validation of mutually exclusive mutational patterns of *NOTCH1* and *PIK3CA*, two significantly mutated genes (SMGs) identified in ESCC via genomic analyses [7-10], and their associations with clinical variables.

## RESULTS

### Mutual exclusivity among SMGs in ESCC

**Figure 1:**
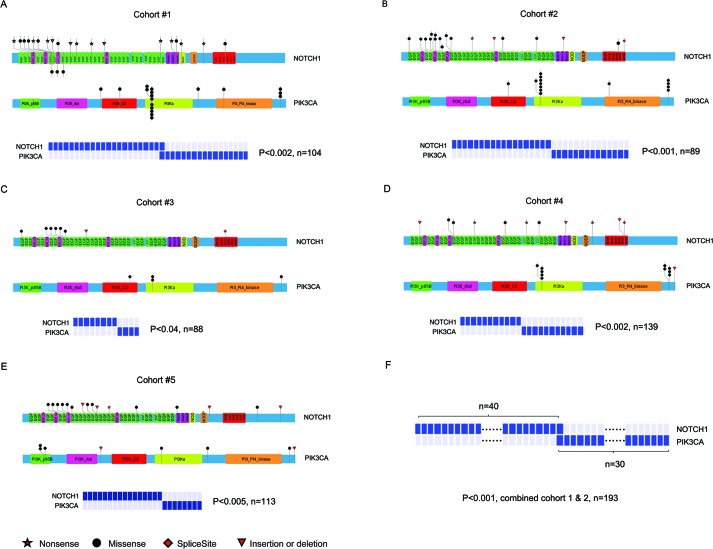
Exclusion between the mutations of *NOTCH1* and *PIK3CA* in ESCC The location of somatic mutations on *NOTCH1* and *PIK3CA* (upper panel) and correlation of these mutations (bottom panel) in cohort #1 **A.**, cohort #2 **B.**, cohort #3 **C.**, cohort #4 **D.**, cohort #5 **E.**, and combined cohort #1 and #2 **F.**. The bottom symbols of *NOTCH1* in Figure 1A represent novel mutations that were identified in our analysis according to the sites reported in catalogue of somatic mutations in cancer (COSMIC). The exclusivity of *NOTCH1* and *PIK3CA* mutations was analyzed via modified model proposed by Ewa Szczurek. A *P* value of <0.05 was considered to be statistically significant.

SMGs that possess a higher mutation rate than the expected background mutation rate may be positive selection during tumorigenesis. Mutations of SMGs are likely to be ‘drivers’ in pathogenesis and these genes may affect the biology of a given tumor [7-10]. We and others previously identified SMGs in ESCC patients, including *TP53, NOTCH1, PIK3CA, FAT1, CDKN2A, FBXW7, ZNF750, AJUBA,* and others [7-10]. We firstly assessed mutational exclusivity among these SMGs as described previously [14]. Most strikingly, an exclusion pattern was observed between the *NOTCH1* and *PIK3CA* mutations in cohort #1 (Figure 1A). Although one patient was found, and further validated via PCR-Sanger sequencing, with concomitant mutations of *NOTCH1* and *PIK3CA*, the frequency of double mutations was 0.0096, which is not significantly different from 0.0 by chi-squared analysis. To further test this observation, we extended the exclusivity analysis to cohort #2 and three other previously published NGS datasets of ESCC patients (cohorts #3 [7], #4 [8], #5 [9]). Even in this expanded series, mutual exclusivity between the *NOTCH1* and *PIK3CA* mutations was found (Figure 1B-1E). When the tumors from cohorts #1 and #2 (n=193), that recruited patients from Taihang Mountains and exhibited similar pattern of *NOTCH1* and *PIK3CA* mutations, were combined, this mutually exclusive pattern between *NOTCH1* and *PIK3CA* mutations was highly significant (Figure 1F). Together, these data suggest that there is a strong inverse relationship between the *NOTCH1* and *PIK3CA* mutations in ESCC that was previously undiscovered.

Additionally, the mutated frequency and mutated sites of these two genes were different among ESCC cohorts. The overall frequency of tumor samples with *NOTCH1* or *PIK3CA* mutations in cohort #1 or #2 was statistically higher than that of cohort #3 or #4 (Supplementary Figure 1B). Moreover, the frequencies of the most common tumor-associated *PIK3CA* mutations, involving either the helical domain (exon 9: c.1624G>A:p.Glu542Lys and c.1633G>A:p.Glu545Lys) or kinase domain (exon 20: c.3140A>G:p.His1047Arg), were significantly different among these cohorts, with 77.8% (14/18) in cohort #1, 85.7% (12/14) in cohort #2, 75% (3/4) in cohort #3, and 25% (2/8) in cohort #5 (Supplementary Figure 1C).

### Clinical and prognostic relevance of *NOTCH1* and *PIK3CA* in ESCC

Tables 1 summarizes the clinicopathological findings for patients in cohort #1 that includes 104 ESCC patients, with a median age of 59.68 years old. The estimated median overall survival (OS) was 40.19 months. Seventy patients received the standard chemotherapy (national comprehensive cancer network clinical practice guidelines in oncology, NCCN guidelines). The remaining 34 patients were treated by surgery alone. Additionally, 89 ESCC patients with a median age of 59.1 years and a median OS of 42.66 months were enrolled in cohort #2. All 89 patients received standard chemotherapy (NCCN guidelines). We then looked for correlations between the *NOTCH1* and *PIK3CA* mutations and clinical parameters. In cohort #1, *NOTCH1* mutations were significantly associated with well-differentiation (*P* = 0.001) and an absence of regional lymph node metastases (N0, *P* = 0.002), and were dramatically enriched in stage I tumors (*P* = 0.011, Table 1). We then validated this analysis in cohort #2. The association of *NOTCH1* mutations with tumor stage and lymph node metastasis also held true in this cohort (*P* < 0.0001, Supplementary Table 2). Furthermore, the relevance of *NOTCH1* mutations to lymph node metastasis was confirmed in our previously published cohort #3 (*P* = 0.011, Supplementary Table 3) [7]. Collectively, our results strongly suggest that the *NOTCH1* mutations were associated with ESCC metastasis; ESCC patients who harbor *NOTCH1* mutations show less risk of metastasis. However, *PIK3CA* mutations were not correlated with clinicopathological characteristics, including tumor differentiation, pathologic stage, and lymph node metastasis.

**Table 1:** Summary of clinical characteristics of ESCC patients with *NOTCH1* and *PIK3CA* mutations in cohort #1

Clinical, epidemiological or pathological feature		NOTCH1				PIK3CA			
		Total(N)	Mutant	Wild-type	*P* value	Total(N)	Mutant	Wild-type	*P* value
All cases		104	22	82		104	17	88	
Age	<60	50	8	42	0.302	50	5	45	0.199
	60-69	40	12	28		40	7	33	
	≥70	14	2	12		14	4	10	
Sex	Male	104				104			
	Female	0				0			
Tobacco use	Yes	96	19	77	0.467	96	15	81	1
	No	8	3	5		8	1	7	
Alcohol consumption	Yes	63	11	52	0.253	62	9	53	0.766
	No	41	11	30		42	7	35	
Family cancer history	Yes	17	5	12	0.557	17	2	15	1
	No	87	17	70		87	14	73	
Tumor location	Upper thoracic	4	0	4	0.027	4	3	1	0.006
	Middle thoracic	42	4	38		42	3	39	
	Lower thoracic	57	17	40		57	10	47	
Histological grade	Grade 1	59	20	39	0.001	59	10	49	0.328
	Grade 2	33	0	33		33	6	27	
	Grade 3	12	2	10		12	0	12	
Pathologic Stage	I	60	18	42	0.011	51	8	43	0.933
	II								
	III	44	4	40		53	8	45	
Pathologic T stage	T1	4	3	1	0.004	4	0	4	0.604
	T2	23	6	17		23	5	18	
	T3	72	13	59		72	10	62	
	T4	5	0	5		5	1	4	
Pathologic N stage	0	54	18	36	0.002	54	9	45	0.706
	1	50	4	46		50	7	43	
Local recurrence	Yes	39	12	27	0.063	39	3	36	0.092
	No	56	10	46		65	13	52	
Chemotherapy treatment	Failure	39	11	28	0.253	39	3	36	0.023
	effective	31	5	26		31	10	21	
	No treatment	24	4	20		24	3	21	
	missing	10	1	9		10	0	10	
Prognosis (Log-rank Mantel-Cox test)	Dead	45	8	37	0.518	45	4	41	0.048
	Survival	41	11	30		41	12	29	
	Missing	18	3	15		18	0	18	
	Median (months)		60	80			47.5	24	

Note: The ESCC cases collected for this study were staged using the NCCN guidelines.

**Figure 2:**
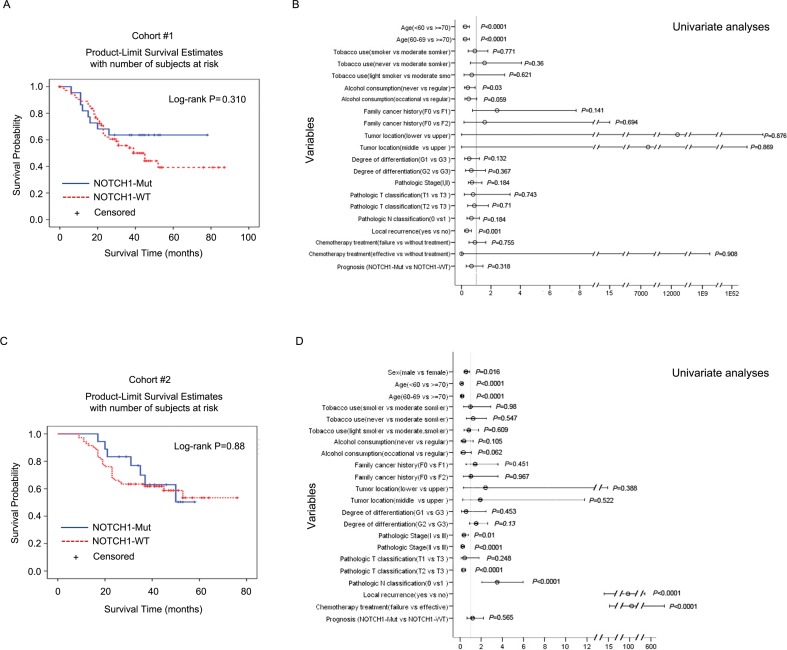
Overall survival among cohorts #1 and #2 according to *NOTCH1* mutation status Kaplan-Meier survival curves for patients with *NOTCH1* mutations or wild-type in cohort #1 **A.** and cohort #2 **C.**. The *P* values were computed by log-rank test. Cox regression analyses were used to adjust for traditional prognostic factors in cohort #1 **B.** and cohort #2 **D.**.

**Figure 3:**
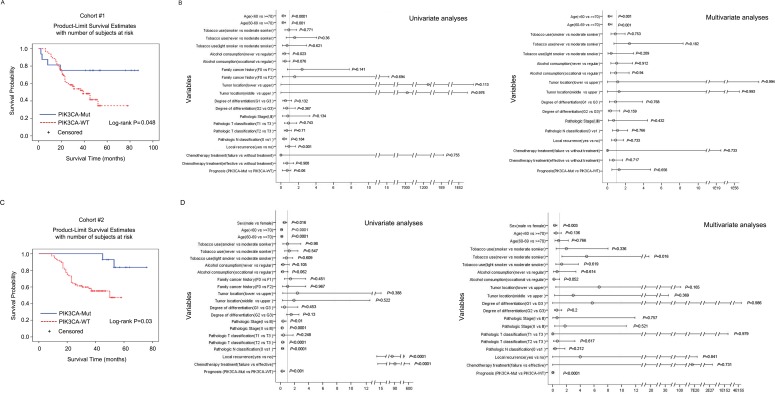
Overall survival among cohorts #1 and #2 according to *PIK3CA* mutation status Kaplan-Meier survival curves for patients with *PIK3CA* mutations or wild-type in cohort #1 **A.** and cohort #2 **C.**. The overall survival data were analyzed using log-rank test, and Cox regression analyses were used to adjust for traditional prognostic factors in cohort #1 **B.** and cohort #2 **D.**.

Next, we used Kaplan-Meier analysis to assess the impact of *NOTCH1* or *PIK3CA* mutations on OS in cohort #1 and validated it in cohort #2. The patients showed a median OS of 60 months and 80 months for the *NOTCH1*-mutated and *NOTCH1*-wild type (WT) groups, respectively, in cohort #1 (Table 1). Surprisingly, although ESCC patients with *NOTCH1* mutations showed less risk of metastasis, the association of *NOTCH1* mutations with OS was not statistically significant (Log-rank (Mantel-Cox), *P* = 0.310, Figure 2A; Cox regression analysis, *P* = 0.318, Figure 2B). This observation was further validated in cohort #2 (Cox regression analysis, *P* = 0.565, Figure 2D). We did, however, find a significant effect on OS for the *PIK3CA* mutations. The *PIK3CA* mutations showed a positive correlation with OS (log-rank *P* = 0.048) in cohort #1 (Figure 3A). This trend reached statistical significance in cohort #2 (Cox regression analysis, *P* < 0.001, adjusted hazard ratio (HR) = 0.031, 95% confidence intervals (CI): 0.006-0.158, Figure 3D) and combined cohort #1 and #2 (P = 0.003, adjusted HR = 0.241, 95% CI: 0.094-0.622, Supplementary Figure 2E). These data indicate that the *PIK3CA* mutation may be a marker of favorable prognosis for ESCC patients from the population in Taihang Mountains, Northern China.

**Figure 4:**
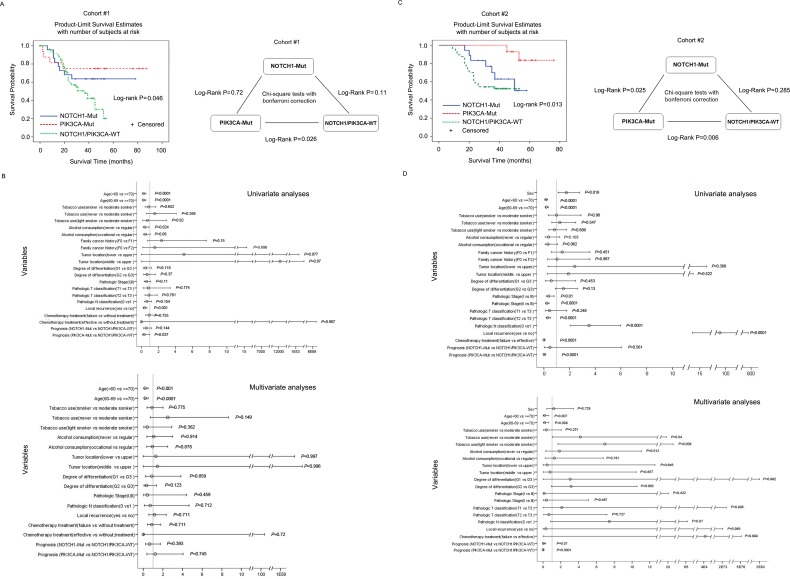
Overall survival among cohorts #1 and #2 according to *NOTCH1* and *PIK3CA* mutation status (A and C) Left panel: Kaplan-Meier survival curves for three groups of patients defined by *NOTCH1* and *PIK3CA* mutations in cohort #1 **A.** or cohort #2 **C.**. The log-rank test was used to calculate the significance. Right panel: chi-square test with Bonferroni correction was used to analyze the OS for the patients in the subgroups. (B and D) Cox regression analysis was used to adjust for traditional prognostic factors in cohort #1 **B.** and cohort #2 **D.**.

Based on the mutually exclusivity of *NOTCH1* and *PIK3CA*, ESCC patients could be divided into three groups: patients with *NOTCH1* mutations, patients with PIK3CA mutations, and patients without mutations of either gene (Supplementary Table 4). We extended our survival analysis in these groups and found that patients with *PIK3CA* mutations had a significantly longer OS (median OS of 80.9 months) than patients without mutations in either gene (median OS 40 months) (log-rank *P* = 0.026, Figure 4A; Cox regression analysis, *P* = 0.037, Figure 4B). Notably, although *NOTCH1* mutations were significantly associated with tumor well-differentiation, early pathologic stage, and low lymph node metastasis, patients with *NOTCH1* mutations tended to have an even shorter OS than patients with *PIK3CA* mutations; the OS of patients with *NOTCH1* mutations was not different from that of patients without mutations in these two genes (Cox regression analysis, *P* = 0.144, Figure 4B). A similar trend was observed in cohort #2 (Figure 4C-4D) and combined cohorts #1 and #2 (Supplementary Figure 3). *PIK3CA* mutations correlate with OS (Cox regression analysis, *P* < 0.001, HR=0.002, 95% CI: 0.0-0.038, Figure 4D), whereas *NOTCH1* mutations show no correlation with OS (Cox regression analysis, *P* = 0.561, Figure 4D). Apart from *NOTCH1* and *PIK3CA*, no SMGs were statistically associated with clinical variables. Although *FAM135B* and *EP300* were associated with OS in previous studies [7, 9], we did not observe any such statistically significant association in our cohort.

### Association of *NOTCH1* and *PIK3CA* mutations with chemotherapy response

**Figure 5:**
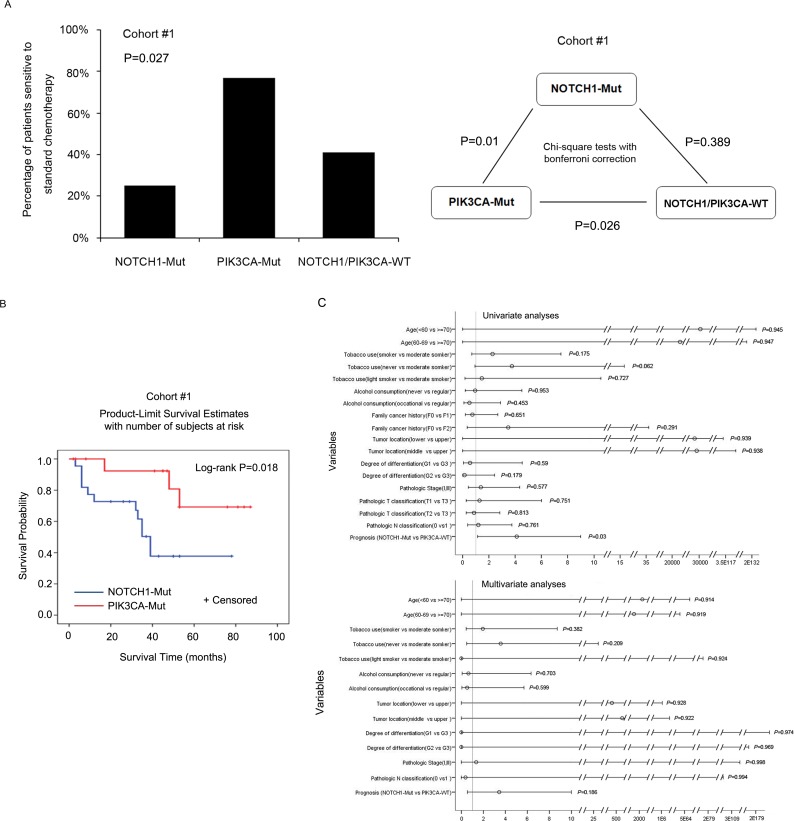
Chemotherapy response and mutation status *of NOTCH1* and *PIK3CA* **A.** Left panel: The frequencies of the patients who exhibited sensitivity to standard chemotherapy are described in three groups: patients with *NOTCH1* mutations, *PIK3CA* mutations, or patients with neither, in cohort #1. Right panel: chi-square test with Bonferroni correction was used to compare the chemotherapeutic efficacy of the subgroups in cohort #1. **B.** PFS of 29 patients with *NOTCH1* or *PIK3CA* mutations in cohort #1. **C.** Cox regression analysis was used to examine the PFS.

In cohort #1, 70 patients received standard chemotherapy, including 16 patients with *NOTCH1* mutations, 12 patients with *PIK3CA* mutations, 1 patient with both the *NOTCH1* and *PIK3CA* mutations, and 41 patients with neither *NOTCH1* nor *PIK3CA* mutations (Supplementary Table 5). Of the 70 patients who received standard chemotherapy, 31 patients responded effectively to chemotherapy; 39 had either tumor progression or tumor recurrence, which were considered to be treatment failures. The median OS of the 70 patients who received standard chemotherapy was 53.48 months. Patients with *NOTCH1* mutations showed a median OS of 27.09 months, whereas those with *PIK3CA* mutations showed a median OS of 80 months. We then evaluated the association between the *NOTCH1* and *PIK3CA* genotype and the efficacy of standard chemotherapy. Of 17 patients who had *NOTCH1* mutations and received standard chemotherapy, 70.6% (12 out of 17) exhibited failure of chemotherapy. Surprisingly, we found no benefit of standard chemotherapy for patients with *NOTCH1* mutations compared to those with mutations in neither gene (*P* = 0.389, Fisher's exact test). Conversely, having a *PIK3CA* mutation was associated with a better response than having mutations in neither gene (*P* = 0.026, Fisher's exact test). Of 13 patients with *PIK3CA* mutations who received standard chemotherapy, only 23% (3 out of 13) showed failure of chemotherapy. Moreover, patients with *PIK3CA* mutations showed significantly better responses than those with *NOTCH1* mutations (*P* = 0.01, Fisher's exact test, Figure 5A, right panel).

We validated this trend in cohort #2, who all received standard chemotherapy treatment. This cohort included 18 patients with *NOTCH1* mutations, 14 patients with *PIK3CA* mutations, and 57 patients mutations in neither gene. Patients in cohort #2 had a median OS of 44.6 months, with a median OS of 32.78 months for patients with *NOTCH1* mutations and 60 months for those with *PIK3CA* mutations. A total of 13 out of 18 patients with *NOTCH1* mutations failed to respond to chemotherapy, whereas 12 out of 14 patients with *PIK3CA* mutations responded to chemotherapy (*P* = 0.001, Fisher's exact test, Supplementary Figure 4A and Supplementary Table 5), consistent with the trend seen in cohort #1.

Next, we examined the effects of *NOTCH1* and *PIK3CA* mutations on the progression-free survival (PFS) of patients in cohorts #1 and #2 who received standard chemotherapy. As expected, patients in cohort #1 with *PIK3CA* mutations showed significantly longer PFS than those with *NOTCH1* mutations by Log-rank (Mantel-Cox) (*P* = 0.018, Figure 5B) and univariate analyses (*P* = 0.03, HR = 4.124, 95% CI: 1.145-14.86, Figure 5C). A similar trend was observed in cohort #2 (Supplementary Figure 4B-C). Our data suggest that patients with *NOTCH1* mutations, who are likely to exhibit a better outcome without chemotherapy, might suffer unnecessarily from side effects of chemotherapy. However, patients with *PIK3CA* mutations could benefit from standard chemotherapy, and could thus survive longer. Therefore, *PIK3CA* mutational status may have a potential role as a biomarker for standard chemotherapy and prognosis in ESCC patients from Taihang Mountains, Northern China.

### *NOTCH1* may act as a tumor suppressor via regulating tumor growth but not metastasis in ESCC

**Figure 6:**
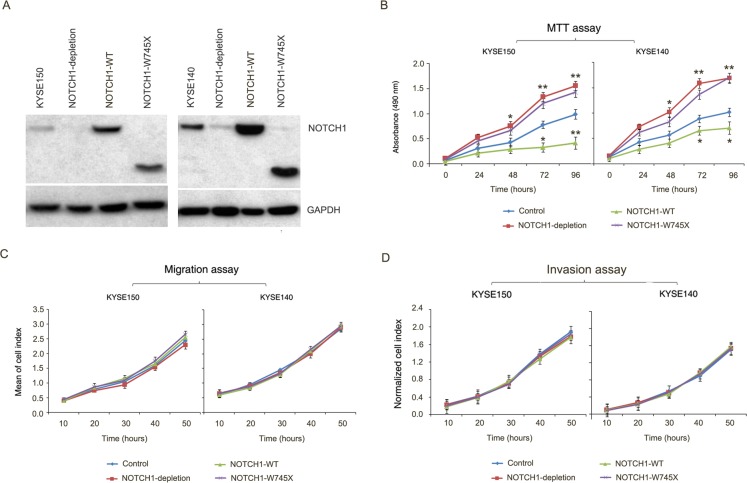
*NOTCH1* acts as a tumor suppressor gene in ESCC **A.** Knockdown of endogenous *NOTCH1* in KYSE150 and KYSE140 cells and subsequent over-expression of *NOTCH1* wild-type or W745X mutant were demonstrated by Western Blotting. GAPDH was used as loading control. **B.** Growth assay shows promoting of proliferation by knockdown *NOTCH1* in KYSE150 and KYSE140 cells. Overexpression of NOTCH1 wild-type but not W745X mutant significantly suppressed cell proliferation. Data are mean ± SD; each experiment was performed in triplicate. **P < 0.01; *P < 0.05. **C.**-**D.** Knockdown of *NOTCH1* or subsequent over-expression of *NOTCH1* wild-type or W745X mutant does not affect migration/invasion of KYSE150 and KYSE140 cells. The data are mean ± SD; each experiment was performed in triplicate.

In general, abnormalities of poor prognostic factors are found in more malignant and advanced cancer cells [1]. However, in our study, we found frequent *NOTCH1* mutations in early-stage malignancy and less metastasis to regional lymph nodes, but with poor prognosis in ESCC patients. In China, patients with ESCC medically unfit for surgery if the tumor was relapsed. Therefore, we had very rare relapsed tumors to analyze *NOTCH1* mutation status. Alternatively, we performed mutation analysis of *NOTCH1* in regional metastatic lymph nodes from 37 of stage III ESCC patients in cohort #1 and 19 of stage III ESCC patients in cohort #2. We found none of *NOTCH1* mutations in these regional metastatic lymph nodes, further supporting that *NOTCH1* mutations associated with early stage and non-lymph node metastasis. Furthermore, we performed functional analysis of NOTCH1-W745X, which located in the epidermal growth factor (EGF) like ligand-binding domain. In light of previous literatures, functional studies have shown that *NOTCH1* gene suppress proliferation and promote differentiation of keratinocytes [15]. Similarly, conditional *NOTCH1*-knockout (mice) develop cutaneous epithelial tumors, and transgenic mice expressing a pan-*NOTCH1* inhibitor develop cutaneous squamous cell carcinomas [16-17]. The tumor-suppressive role for *NOTCH1* in squamous cells is also supported by recent sequencing studies of related tumor types, such as squamous cell carcinomas of the head and neck, skin, and lung [15, 18-19]. Consistently, a tumor-suppressive role for *NOTCH1* in ESCC was supported by MTT assay in KYSE150 and KYSE140 cells (Figure 6A-6B). However, we observed no correlation of *NOTCH1* gene with cell migration and invasion as monitored by the iCELLigence RTCA DP system (Figure 6C-6D), indicating that *NOTCH1* may involve cell proliferation but not migration and invasion in ESCC cells. Together with our genetic observations, these functional data indicate that *NOTCH1* may act as a tumor suppressor via regulating tumor growth but not metastasis in ESCC.

## DISCUSSION

A striking finding of this study is the previously unreported, significant mutually exclusive mutational pattern between *NOTCH1* and *PIK3CA* in ESCC. Moreover, our results suggest that patients who harbor *NOTCH1* mutations have a statistically significant association with well differentiation, an early stage of malignancy, and less regional lymph node metastasis; however, they exhibited a poor outcome and failure to respond to standard chemotherapy treatment. In contrast, patients who harbor *PIK3CA* mutations showed a better response to standard chemotherapy and exhibited favorable survival. Recently, the systematic analysis of the Cancer Genome Atlas (TCGA) Pan-Cancer mutation data set across 12 major cancer types revealed 14 mutually exclusive pairs among 127 SMGs, including pairs of TP53 and *PIK3CA* with significant exclusivity in breast adenocarcinoma [12]. However, mutual exclusivity between *NOTCH1* and *PIK3CA*, which was identified as SMGs via genomic analyses of ESCC patients, has not previously been implicated in any type of cancer [7-10, 12].

In this study, we observed different patterns of SMGs, especially different distributions of mutation frequencies and mutated sites of *NOTCH1* and *PIK3CA* between ESCC patients from Northern China and those from Southern China. The frequencies of *PIK3CA* and *NOTCH1* mutations in cohorts #1 and #2 (from Taihang Mountain, Northern China) were higher than those of cohorts #3 (from Chaoshan District, Southern China [7]) and cohorts #4 and #5 (recruited from CICAMS, where patients came from all over the country and did not have a limited geographic distribution pattern [8, 9]). The possible reasons for this pattern could be genetic and environmental etiologic factors [6, 12]. In the high-risk areas of China, there is a strong tendency toward familial aggregation of ESCC, which suggests that genetic susceptibility in combination with exposure to environmental risk factors contributes to the high rates of ESCC in these areas [20]. In addition to ethnicity, other possible etiologic factors such as lifestyle patterns, nutritional deficiencies, and mycotoxin-contaminated foods could play important roles in these high-risk areas [21-24]. Therefore, the different environmental carcinogens between northern and southern China might cause different genetic diversity (e.g., mutational pattern of SMGs) in ESCC individuals from the Taihang Mountains and Chaoshan area.

Patients diagnosed at early stage of cancer progression may exhibit better survival over time [25]. *NOTCH1* was mutated in around 20% out of ESCC patients, and correlated with well differentiation, early TNM stage, and absence of regional lymph node metastases. The close link between *NOTCH1* mutation types and clinicopathological features leads us to speculate that ESCC patients with *NOTCH1* mutations may have a better prognosis. However, we did not observe such correlation in our patients. Meanwhile, a worse response to standard chemotherapy was observed on patients with *NOTCH1* mutations compared to those with *PIK3CA* mutations. Patient outcomes may correlate with the clinical stage of the cancer at diagnosis and the status whether or not patient has received chemotherapy [26]. However, while chemotherapy remains the backbone of current cancer treatment, it is limited by a narrow therapeutic index, significant toxicity leading to severe side effects and frequent acquired resistance [27]. Considering that *NOTCH1* mutations associated with early stage and non-lymph node metastasis that was supporting by both genetics alterations and functional study, however, no statistically significant differences in patient outcome and standard chemotherapy benefit were observed in patients contained *NOTCH1* mutations, we speculate that there is a possibility that patients harbor N*NOTCH1* mutations who should exhibit a better outcome may suffer side-effect of chemotherapy or receive extra treatment. These obstacles call for novel therapeutic approaches for these patients.

Recently, the role of *NOTCH* signaling in cancers has been reported, with *NOTCH* signaling having both oncogenic and tumor-suppressive roles, depending on the cellular context [20]. In our and others' cohorts of ESCC patients, many of the missense mutations in the *NOTCH1* gene occurred at or near identified important domains, such as the ligand-binding domain (EGF repeats) and the majority of the mutations were predicted to alter the protein N-terminal to the transmembrane region. Moreover, the nonsense mutations (e.g. c.2234G>A:*p*.Trp745*) observed in *NOTCH1* gene generate a premature stop codon, which results in a truncated NOTCH1 protein that lacks the C-terminal domain, which contains a proline-glutamate-serine-threonine (PEST) sequence and is important for transcription activation. Thus, although the activating mutations in *NOTCH1* were identified in T-cell acute lymphoblastic leukemia, chronic lymphoblastic leukemia, and breast cancer [21-23], the pattern of the mutations in genes that involve the *NOTCH* pathway suggests its potential tumor suppressing roles in ESCC, as has been reported for head and neck squamous cell carcinoma, chronic myelomonocytic leukemia, and lung squamous cell carcinoma [20, 24]. This interpretation is consistent with the functional studies of the role of *NOTCH1* in ESCC cells, as *NOTCH1* depletion promotes tumor cell proliferation in tissue culture. Inhibition of *NOTCH1* pathway has been shown to sensitize cancer cells to chemotherapy in prostate cancer, ovarian cancer, colon cancer, and glioma [28-31]. Recent studies have aimed to develop antibodies against specific *NOTCH* receptors and ligands with the hope of limiting side effects while providing the same therapeutic benefit as gamma secretase inhibitors (drugs that inhibit *NOTCH* signaling); these studies were carried out in human cancers with commonly overactivated mutations in *NOTCH1* that confer a survival advantage on the tumors, leading to poorer outcomes for the patients [15, 27]. However, owing to the potential tumor suppressing roles rather than the oncogenic function of *NOTCH1* in ESCC, targeting *NOTCH* signaling might not be helpful for treating ESCC.

In accordance with the previous literature [32, 34], the *PIK3CA* mutation could serve as a favorable predictive biomarker in ESCC patients from Taihang Mountains, Northern China. However, *PIK3CA* mutations were not associated with patient outcomes in ESCC patients from the Chaoshan population, Southern China (cohort #3) and from CICAMS, which does not have any geographic distribution limitations (cohort #5) [7, 9]. Possible reasons for the conflicting results include the mutation frequency, mutation sites of *PIK3CA*, or perhaps the sample size of the cohorts. In addition to the different mutation frequencies of *PIK3CA* among the cohorts, the most common tumor-associated *PIK3CA* mutations (those involving the helical domain (exon 9: c.1624G>A:p.Glu542Lys and c.1633G>A:p.Glu545Lys) or the kinase domain (exon 20: c.3140A>G:p.His1047Arg)) had significantly different frequencies among these cohorts. In previous studies, the c.1633G>A:p.Glu545Lys and c.3140A>G:p.His1047Arg mutations have been implicated in favorable overall survival in the ESCC patients [33, 34]. *PIK3CA* has been named as the key oncogenic effector and could be a potential driver mutation in tumorigenesis of ESCC [35] and these mutants have previously shown oncogenic effects *in vivo* and may act as a p otential target site of treatment as in other cancer types[36]. We speculate that the lower frequency of mutations in *PIK3CA* hotspots in cohort #5 results in a negative association between *PIK3CA* mutations and survival. Moreover, our previous mutational signature analysis showed that hotspot mutations (c.1624G>A:p.Glu542Lys, c.1633G>A:p.Glu545Lys) of *PIK3CA* were significantly enriched in ESCC tumors that had an apolipoprotein B mRNA-editing enzyme catalytic (APOBEC) signature in both cohort #1 and cohort #3, which implicates APOBEC activity as a common key driver of *PIK3CA* mutagenesis in ESCC patients from Northern or Southern populations in China [10]. Therefore, the effect of *PIK3CA* mutations on survival prognosis might be observed when cohort #3 is extended to increase the sample size.

Together with previously reported NGS data, our results document for the first time that *NOTCH1* and *PIK3CA* mutations are mutually exclusive alterations in ESCC. Although *NOTCH1* mutations had a statistically significant association with well-differentiation, early stage of malignancy and less regional lymph node metastasis in ESCC, patients who harbor *NOTCH1* mutations would not benefit from standard chemotherapy, and alternative therapeutic strategy must be developed for these patients. Conversely, patients who harbor *PIK3CA* mutations could benefit from standard chemotherapy, and thus *PIK3CA* mutational status may have a potential role as a biomarker for standard chemotherapy and prognosis. These results raise the possibility for the categorization of ESCC using the mutations of *NOTCH1* and *PIK3CA*. However, the *NOTCH1* and *PIK3CA* mutated samples represented a small proportion of ESCC, and the majority of samples had wild-type *NOTCH1* and *PIK3CA*. Further studies of larger patient cohorts including all stages ESCC from multiple restricted areas with high incidences of ESCC will be necessary to validate the mutual exclusivity of *NOTCH1* and *PIK3CA* and the relationship of these two genes with clinical features, as well as to further refine the potential subclassification of ESCC based on these genetic alterations. Whether they indeed reflect the biology of ESCC and the patients' clinical course remains to be seen. The results could extend our knowledge to potential molecular diagnosis markers, predicting overall patient survival and chemotherapy response in a portion of ESCC patients, which could guide the therapeutic strategy to combat ESCC.

## MATERIALS AND METHODS

### Ethics approval

This study was approved by the ethical committee of the Shanxi and Henan, China (IRB of Shanxi Medical University, Approval No. 2009029, and the Ethics Committee of Henan Cancer Hospital, Approval No. 2009xjs12). All samples were obtained before treatment according to the guidelines of the local ethical committees, and written informed consent was received from all participants prior to inclusion in the study.

### Patient characteristics

A total of 104 ESCC patients from the Taihang Mountains were recruited as described previously [10]. Pairs of tumors and matched normal tissues from 14 patients underwent whole-genome sequencing (WGS, median coverage of 65×), and 90 pairs of samples underwent whole-exome sequencing (WES, median coverage of 132×) [10]. Additionally, to provide high-confidence mutations, 96 pairs of tumors and matched normal tissue were selected for deep target capture-based validation (TCS, at least 365×). A detailed description of next-generation sequencing analysis pipeline is provided in the Online Methods. All subsequent analyses relied on these validated data and not on the primary genome discovery sequence. A summary of clinical characteristics of the analyzed patients (cohort #1) is presented in Table 1 and Supplementary Table 1. In this cohort, seventy patients received standard chemotherapy (NCCN guidelines). Most of the patients had undergone platinum based chemotherary conbined with 5-fluorouracil (FU) or paclitaxel. Patients were treated with paclitaxel on the first day combined with platinum on day 1 or devided into 4-5 days. 5-FU was used from day 1 to day 5 or continuous infusion combined with platinum as above. A small part of patients used concomitant chemotherapy was continuous infusion of 5-FU alone with a dose of 300 mg/m2/day during the whole course of treatment. Treatment cycles were repeated every 3-4 weeks (Supplementary Table 5). Overall survival (OS) data were available for 95 patients. We examined the prevalence of overall failure of standard chemotherapy in cohort #1, with treatment failure as either occurrence of cancer-related death or emergence of recurrent tumors in patients who received standard chemotherapy.

We further validated our results in an extended ESCC cohort (cohort #2). A total of 89 ESCC patients from Shanxi and Henan provinces, Taihang Mountains received standard chemotherapy (NCCN guidelines) and were randomly assigned to cohort #2 (Supplementary Table 1-2). The genotype of *NOTCH1* and *PIK3CA* was analyzed in 89 formalin-fixed paraffin-embedded tumors by PCR-Sanger sequencing using the paired primers 1F (ACCCGATGCGGTTAGAGCC), 1R (ACACAATAGTGTCTGTGACTCC), 2F (ACTCCATGCTTAGAGTTGGAG), and 2R (GGATTGTGCAATTCCTATGCAATC) for PIK3CA and 1F (GTGACTGCTCCCTCAACTTCAAT), 1R (CTGTCACAGTGGCCGTCACT), 2F (GTCAACGCCGTAGATGACCT), and 2R (TCTCCTCCCTGTTGTTCTGC) for *NOTCH1*. Moreover, we validated our result in another three independent ESCC mutation datasets: Cohort #3 from our previous report of 17 WGS and 71 WES samples, which was recruited from the Chaoshan District of Guangdong Province, another area that has high ESCC prevalence in China [7]. Cohort #4 was from Lin *et al.* of 20 WES and 119 TCS samples, which were recruited from CICAMS and Linxian Cancer Hospital [8]. Cohort #5 from Gao et al. contained 113 WES samples, which were recruited from CICAMS [9].

### Mutually exclusive pattern

The mutual exclusivity pattern was detected as described previously [14]. Briefly, n columns correspond to *NOTCH1* and *PIK3CA* mutations and m rows correspond to patients whose tumor samples were collected (with m n). For each patient and gene, the dataset records a binary alteration status of the gene observed in the patient, with 0 standing for absence and 1 for presence of alteration. We define the set of model parameters θ={γ,δ,α,β} with coverage γ, impurity δ, false positive rate α and false negative rate Δ; and a set of random variables including variable C that indicates patient coverage, variable H that specifies the single exclusively mutated gene in a covered patient, variable T that represent the true alterations of genes, and variable Y that correspond to the alteration status of genes recorded in the data. The model is defined by: $P(Y_{g}|T_{g}\theta )=\alpha^{Y_{g}(1-T_{g})}{\left(1-\alpha \right)}^{\left(1-Y_{g})(1-T_{g}\right)}\beta^{(1-Y_{g})T_{g}}{\left(1-\beta \right)}^{Y_{g}T_{g}}$. A P value of <0.05 was considered to be statistically significant.

### Statistical analyses

Fisher's exact test was used to analyze categorical features such as the association of *NOTCH1* or *PIK3CA* mutations with clinical and pathological features, the distribution of gene mutations in different clusters or different cohorts, and the response rate to chemotherapy among the subgroups. A *P* value of <0.05 was considered to be statistically significant. Comparisons of binary and categorical patient characteristics between subgroups were performed using the chi-square test with Bonferroni correction. A *P* value of <0.025 was considered to be statistically significant.

The OS and progression-free survival (PFS) distributions were described by the Kaplan-Meier curves, and statistical significance was calculated using the log-rank test. OS was evaluated from the time of diagnosis to death or the final follow-up. Censored cases were defined as patients who lost contact during the follow-up and who were still alive at the end of the study. PFS was evaluated from the time of diagnosis to the occurrence of cancer-related death or emergence of recurrent tumors or the last follow-up. Censored cases were defined as patients who were still alive and had no recurrent tumors at the end of the study. The primary outcome was clinical benefit, which was defined as failure or response to chemotherapy. The occurrence of cancer-related death or the emergence of recurrent tumors in patients who had received standard chemotherapy was considered to represent failure of chemotherapy. Univariate and multivariate analyses with the Cox proportional hazards model were used to examine the association between mutations and overall survival and response to chemotherapy. For multivariate analysis, in addition to the confounders with univariate *P* values of < 0.05, traditional prognostic factors, including age, smoking, drinking, clinical stage, tumor differentiation, and chemotherapy treatment, were adjusted for the analysis of the prognosis.

### *NOTCH1* knockdown and its mutant over-expression

Lentivirus vector pLKO.1-puro and its packaging plasmids pMD2.G and psPAX2 were obtained from Addgene. *NOTCH1* knockdown experiments were performed in two ESCC lines with high endogenous *NOTCH1* expression: KYSE150 and KYSE140. Two independent shRNAs, (5′-UCGCAUUGACCAUUCAAACUGGUGG-3′, Scrambled siRNA 5′-AAAUGUGUGUACGUCUCCUCC-3′; *NOTCH1* or nontargeting ON-TARGET plus SMART siRNAs, Dharmacon RNATechnologies), were cloned into pLKO.1-puro vector as described previously [10]. Infection of retrovirus containing both siRNAs resulted in similar phenotype changes in the pre-experiments (data not shown). Thus, in order to obtain high infection efficiency, we pooled these two independent siRNAs retrovirus to infect KYSE cells. A non-specific targeting shRNA was also cloned into pLKO.1-puro vector using as scrambled control. Relative expression of *NOTCH1* was normalized to GAPDH expression level. For overexpression experiments, *NOTCH1* wild-type and *NOTCH1*-W745X mutant were cloned into pLV-EGFP(2A)-puro-GFP-vector and validated by sequencing. For overexpression experiments, we used pLV-EGFP(2A)-puro-GFP-vector as a control. *NOTCH1*-depleted KYSE cells were infected with viruses as previously described [10]. 24 hours post infection, cells were subjected for subsequent experiments.

### Immunoblotting

Cells were lysed for 30 min in Triton buffer (1% Triton X-100, 50 mM Tris–HCl pH 7.6, 150 mM NaCl, 1% sodium deoxycholate, 0.1% SDS) supplemented with protease and phosphatase inhibitors (1 mM PMSF, 2 mM sodium pyrophosphate, 2 mM sodium betaglycerophosphate, 1 mM sodium fluoride, 1 mM sodium orthovanadate, 10 μg/mL leupeptin and 10 μg/mL aprotinin). Lysates were cleared by centrifugation at 15,000 x g at 4°C for 15 min, and protein concentrations were determined using the Bradford method. Fifty μg of protein were separated by SDS-polyacrylamide gel electrophoresis and transferred onto Immobilon-P membranes. Proteins were detected by using anti-N-terminal NOTCH1 antibody (GENWAY Biotech Inc). Antibody binding was detected using horseradish peroxidase-labelled anti-mouse (Sigma) or anti-rabbit (Cell Signaling) antibodies and chemiluminescence was detected using a LAS4000 device (Fuji). Equal protein loading was confirmed with antibodies against GAPDH (Transgen).

### MTT assay

4×10^3^ cells were seeded in 96-well plates and incubated in normal condition for 24 hours. Cells were treated with 100μl of 5mg/ml of MTT (Invitrogen) solution for 4 hours at 37°C until crystals were formed. MTT solution was removed from each well and 100μl of DMSO was added to each well to dissolve the crystals. Color intensity was measured by Microplate Reader (Bio-Rad) at 490 nm. Each experiment consisted of four replications and at least three independent experiments were carried out.

### Migration/invasion assay

Migration and invasion assays were performed in 16-well CIM plates in an xCELLigence RTCA DP system (ACEA Biosciences) using matrigel basement membrane matrix (BD) for real-time cell migration analysis as described previously [10]. Briefly, 30,000 cells per well were seeded as 5 duplicates in serum-free medium at the upper compartment of the CIM plates coated with or without matrigel. Serum-complemented medium was added to the lower compartment of the chamber, and then started measurement in xCELLigence RTCA DP system and analyzed the CI (Cell Index) curves to determine cell invasion activity. For negative controls, we added serum-free medium at both upper and bottom chambers. The concentration of matrigel was 1:6 for KYSE150 cells and 1:10 for KYSE140 cells. The cell index representing the amount of migrated cells was calculated with the RTCA Software from ACEA Biosciences (San Diego, CA). At least three independent experiments were carried out; for each independent experiment, 5 duplicates were performed for each group.

## SUPPLEMENTARY MATERIAL FIGURES












