# Supplementary material for: Mutually exclusive mutations in NOTCH1 and PIK3CA associated with clinical prognosis and chemotherapy responses of esophageal squamous cell carcinoma in China
Source: Oncotarget. 2015 Oct 29;7(3):3599–613. doi: 10.18632/oncotarget.6120 (PMC4823130; doi:10.18632/oncotarget.6120)
Supplement: Supplementary file 1 [file oncotarget-07-3599-s001.pdf]

Mutually exclusive mutations in *NOTCH1* and *PIK3CA* associated with clinical prognosis and chemotherapy responses of esophageal squamous cell carcinoma in China

Supplementary Material

Target capture resequencing

To provide high-confidence mutations, non-silent mutations, including nonsynonymous mutations, stopgain mutations, stoploss mutations, frameshift deletions, frameshift insertions, nonframeshift deletions, and splice-site mutations identified in the whole-genome and exome sequencing sets were selected for target capture-based validation (TCS). Briefly, non-silent mutations (6873) and small indels (125) in coding regions identified from 96 of the 104 samples were designed on a Nimblegen customized capture array (Roche Company). The remaining 8 samples with few somatic mutations were not included in the TCS validation. Genomic DNAs from 48 tumors of stage I, 48 tumors of stage III, and matched normal tissues were fragmented, and libraries were constructed following the same method with an exome-capture experiment. For SNVs and small indels, we tiled approximately 200 bp targets across the variant of interest, including a minimum buffer of 100 bp in each direction. After library preparation and hybridization, sequencing was performed on an Illumina HiSeq 2000 platform. Each tumor was sequenced to at least a depth of 300×, and the SNVs were called with the same pipeline except that the variant allele frequency was decreased to 5% to guarantee that low-frequency mutations were retained. Somatic indels were manually inspected across 104 normal samples to remove the germline mutations. The mean coverage achieved was 365× in target capture-based sequencing with 354× in tumor and 375× in normal. Validation lanes were aligned to the reference sequence, and BAM files were created in the same manner as described above. Significantly mutated genes (SMGs) were identified using MutSigCV (freely available for noncommercial use at http://www.broadinstitute.org/cancer/cga/mutsig), as previously described. The validation rates were 97.8% for identified single-nucleotide variations (SNVs). All subsequent analyses relied on these validated data and not on the primary genome discovery sequence.

Data-sharing statement

The data, including sequence data and analyses, are available for download from the database of the Sequence Read European Genome-phenome Archive under accession no. EGAS00001001487 https://www.ebi.ac.uk/ega/studies/EGAS00001001487

Supplementary Figure 1

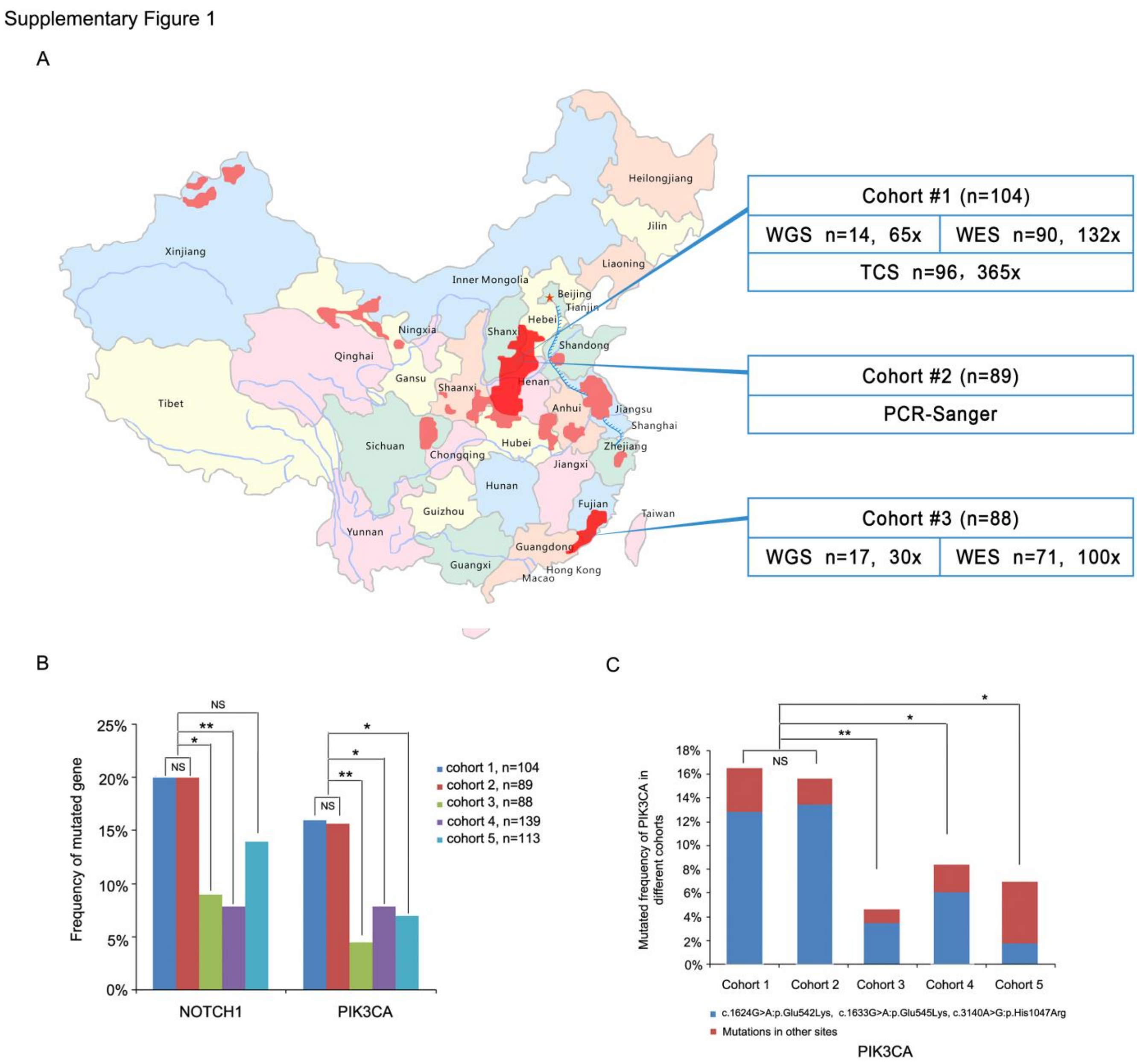

Supplementary Figure 1. The cohort information and the mutated frequency of *NOTCH1* and *PIK3CA*. **(A)** Patients from specific populations in cohorts #1, #2, and #3 and their calculations of the coverage. The red color in the map represents high-risk area of ESCC in China. **(B)** Comparison of the mutation frequency of *NOTCH1* and *PIK3CA* in different cohorts. **(C)** Comparison of the distribution of tumor-associated *PIK3CA* mutations that involve either helical domain (exon 9: c.1624G>A:p.Glu542Lys, c.1633G>A:p.Glu545Lys) or kinase domain (exon 20: c.3140A>G:p.His1047Arg) and other sites. Fisher's exact test was used to calculate statistical significance. NS denotes without significance; **P*<0.05, ** *P*<0.01.

Supplementary Figure 2

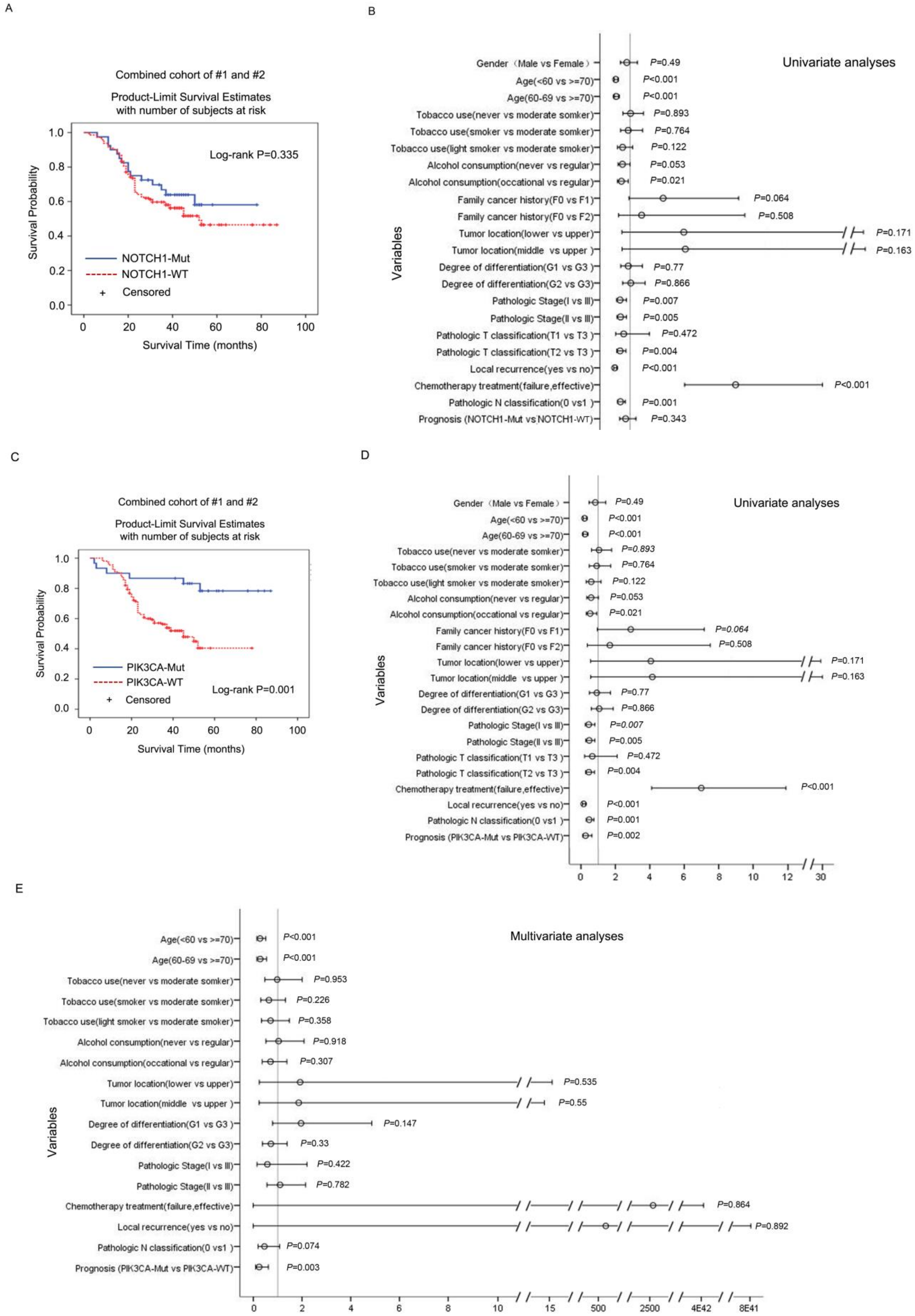

Supplementary Figure 2. Overall survival among combined cohort #1 and #2. **(A** and **C)** Kaplan-Meier survival curves for the patients with *NOTCH1* mutations (A) or *PIK3CA* mutations (C). The overall survival data were analyzed using the log-rank test, and Cox regression analyses were used to adjust for the traditional prognostic factors (**B**-**E**).

Supplementary Figure 3

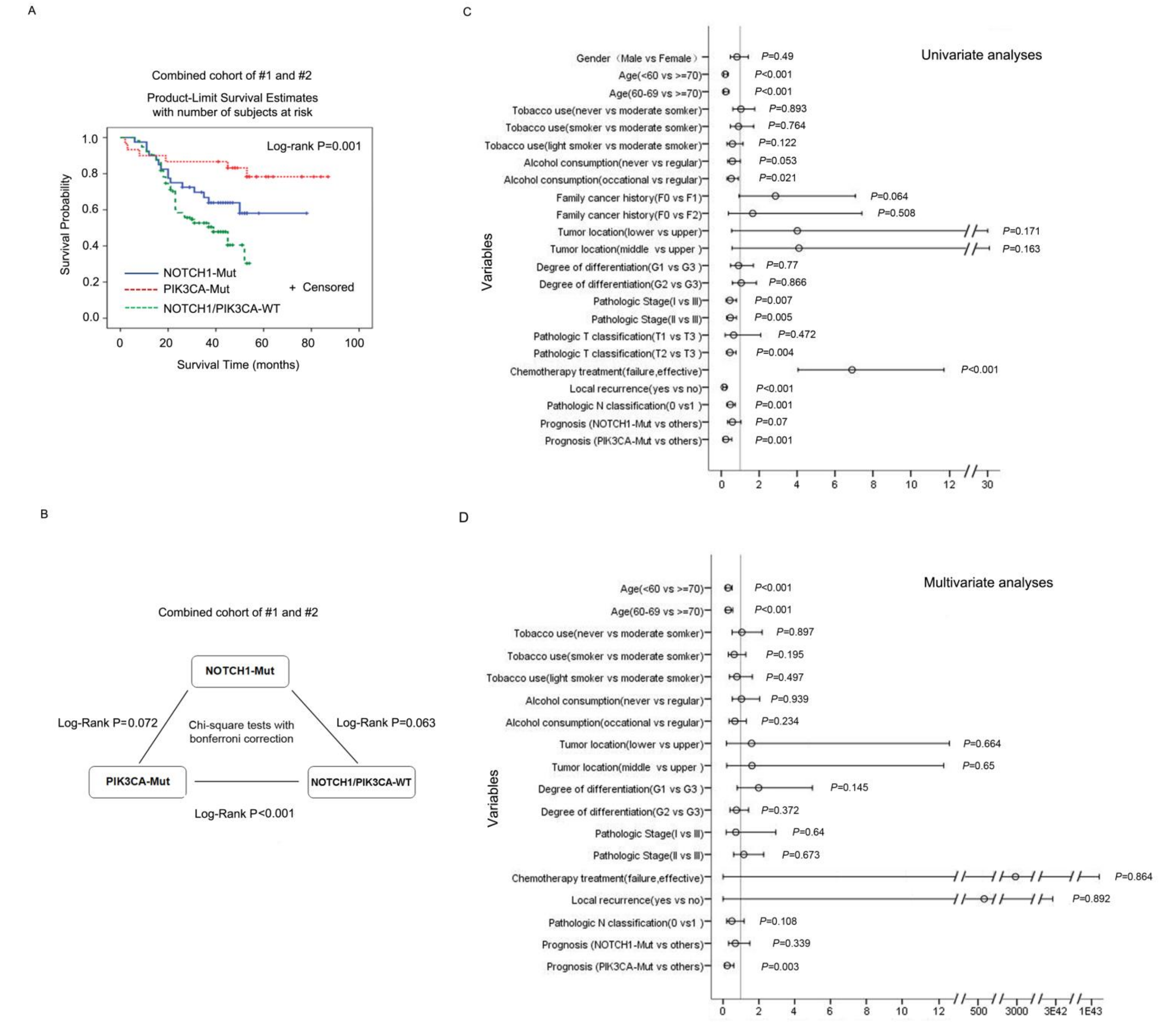

Supplementary Figure 3. Overall survival amongcombined cohort #1 and #2 according to *NOTCH1* and *PIK3CA* mutation status. **(A)** Kaplan-Meier survival curves for three groups of patients defined by *NOTCH1* and *PIK3CA* mutations in combined cohort #1 and #2. The log-ranktest was used to calculate the significance. **(B)** Chi-square test with Bonferroni correction was used to analyze the OS for the patients in the subgroups. **(C-D)** Cox regression analyses were used to adjust for traditional prognostic factors in this combined cohort.

Supplementary Figure 4

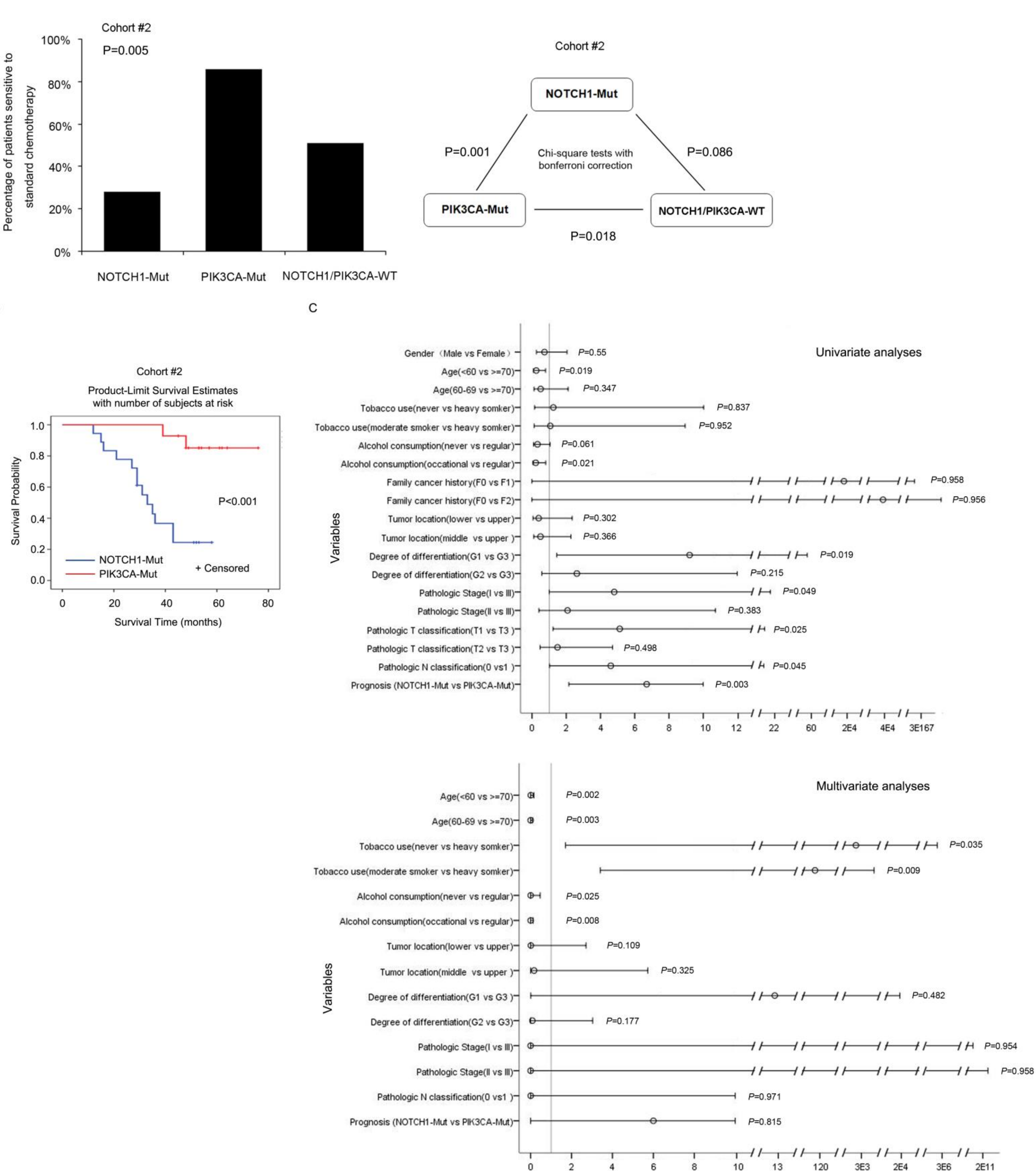

Supplementary Figure 4. Chemotherapyresponse relevance of the *NOTCH1* and *PIK3CA*

mutation status in cohort #2. **(A)** Left panel: Interaction between *NOTCH1* or *PIK3CA* genotypes and the efficacy of standard chemotherapy. The frequency of patients who exhibited sensitivity to standard chemotherapy in three groups (patients with *NOTCH1* mutations, *PIK3CA* mutations, or neither), in cohort #2. A chi-square test was used to analyze the statistical differences. Right panel: chi-square-test withBonferroni correction was used to compare the chemotherapeutic efficacies of the subgroups in cohort #2. **(B)** Progression-free survival of patients with *NOTCH1* or *PIK3CA* mutations in cohort #2. The cases with *PIK3CA* mutations showed a tendency toward a longer survival (P<0.001, log-rank test). **(C)** Cox regression analyses were used to examine the PFS.

Supplementary Figure 5

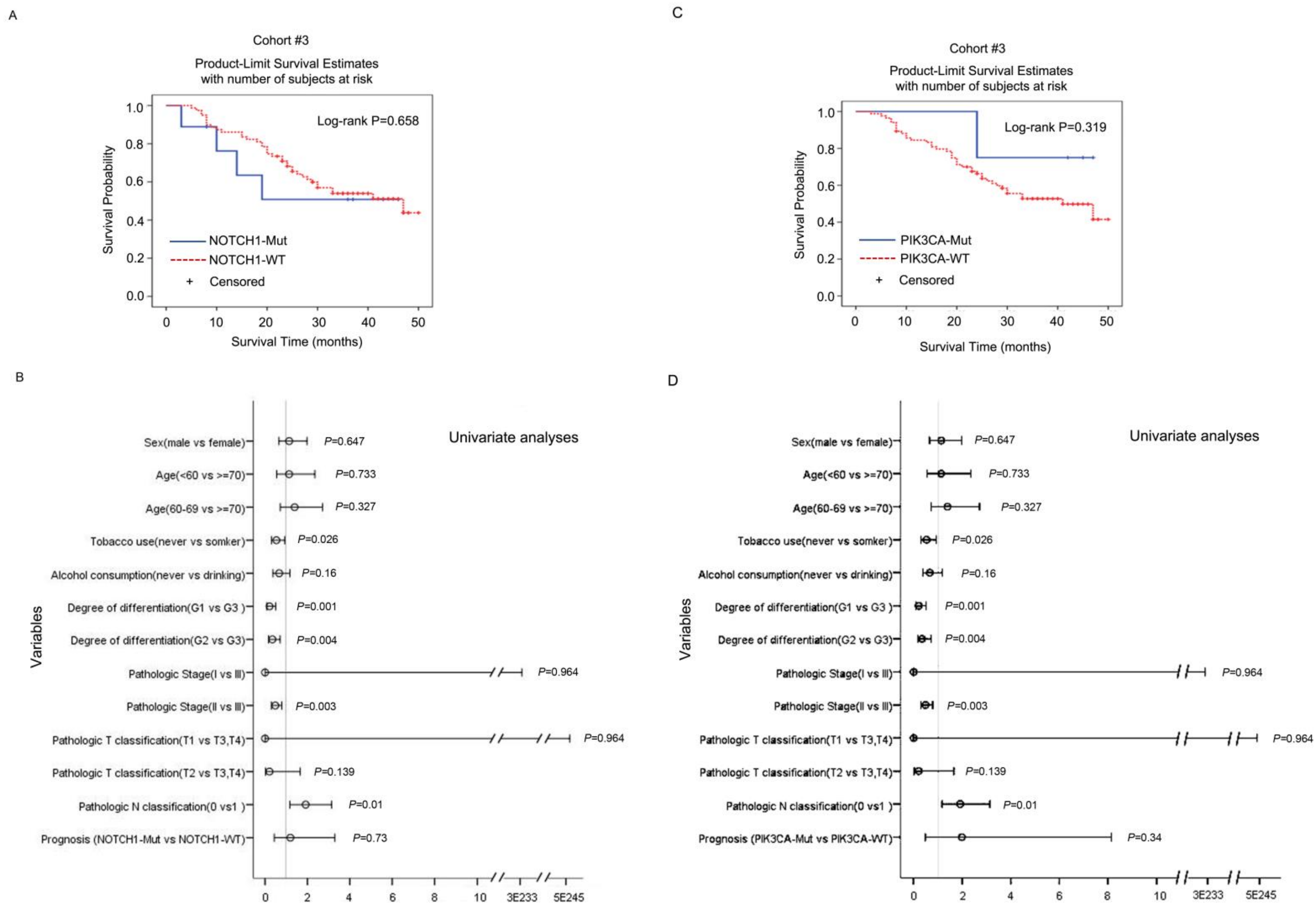

Supplementary Figure 5. Overall survival among cohort #3. **(A** and **C)** Kaplan-Meier survival curves for the patients with *NOTCH1*mutations (A) or *PIK3CA* mutations (C). The overall survival data were analyzed using the log-rank test. (**B** and **D**) Cox regression analyses were used to adjust for the traditional prognostic factors.
